# Modelling Broiler Requirements for Lysine and Arginine

**DOI:** 10.3390/ani11102914

**Published:** 2021-10-09

**Authors:** Bernardo Rocha Franco Nogueira, Nilva Kazue Sakomura, Matheus de Paula Reis, Bruno Balbino Leme, Marie-Pierre Létourneau-Montminy, Gabriel da Silva Viana

**Affiliations:** 1 Department of Animal Sciences, Faculty of Agrarian and Veterinary Science, São Paulo State University, Jaboticabal 14884-900, São Paulo, Brazil; bernardorfnogueira@gmail.com (B.R.F.N.); nilva.sakomura@unesp.br (N.K.S.); matheusdpreis@gmail.com (M.d.P.R.); bruno-balbino@hotmail.com (B.B.L.); 2 Department of Animal Sciences, Université Laval, Québec City, QC G1V 0A6, Canada; marie-pierre.letourneau-montminy.1@ulaval.ca; 3 Production Systems, Natural Resources Institute Finland (Luke), 31600 Jokioinen, Finland

**Keywords:** amino acids, efficiency of utilization, factorial approach, dilution technique

## Abstract

**Simple Summary:**

Achieving a close balance between amino acids provided by feeds and the estimated requirements of broilers is crucial to increase the economic and environmental efficiency in intensive poultry rearing. The utilization of factorial models to predict amino acid requirements requires previous knowledge of the efficiency with which such amino acids are utilized for growth. This study aimed to investigate broiler responses to lysine (Lys) and arginine (Arg) and, based on such responses, to estimate the efficiency with which birds utilize both amino acids for growth. Once estimated, the efficiency values were used to develop two factorial models to predict broiler intakes of Lys and Arg. Overall, the utilization efficiencies were calculated as 0.79 and 0.62 for digestible lysine and arginine intake, respectively. Based on such efficiencies, the male and female broiler requirements for digestible lysine and arginine to deposit 1 g of body protein were 94.9 and 92.9 mg, respectively.

**Abstract:**

Six assays were conducted to investigate male and female broiler responses to standardized ileal digestible (SID) lysine (Lys) and arginine (Arg). Response data were modeled to estimate the efficiency of utilization (*k*) of both amino acids and adjust factorial models to predict bird intake for SID Lys and SID Arg. In each assay, 1280 Cobb 500^®^ broilers (640 male and 640 female) were randomly assigned to one of sixteen dietary treatments with four replicates of 20 birds. Dietary treatments consisted of crescent levels of SID Lys or SID Arg based on the dilution technique. The values of *k* determined for each phase (1- to 14-, 15- to 28-, and 29- to 42-d-old) and sex were contrasted using linear regression with groups (sex and phase). The estimated efficiencies were 0.79 for Lys and 0.62 for Arg, which were unaffected by phase or sex. Factorial models based on body weight and weight gain (M1) and on body and feather protein weight and deposition (M2) were applied to estimate the SID Lys and Arg intake for growth. The amino acid intake based on M2 had a lower error of prediction. Broiler chickens require 94.9 and 92.9 mg/d of SID Lys and SID Arg to deposit 1 g of body protein.

## 1. Introduction

Conventionally, broiler nutritional needs have been estimated by empirical mathematical models (linear response plateau, curvilinear response plateau, exponential, etc.), which describe amino acid (AA) requirements as optimal doses required to optimize performance and carcass responses [1]. Although widespread, such an approach has some limitations, which might affect the accurate representation of bird requirements [2]. First, by fitting broiler responses to such models, a single optimal dose is estimated for a given growth period, which, therefore, characterizes requirements as static for the entire growth phase. The misconception of such an interpretation is that birds are never in a steady state; rather, their body protein accretion and body chemical composition change dynamically as they grow [3], which consequently affect their nutritional requirements for AAs. Second, the estimates obtained from such models represent strictly the experimental conditions under which birds were reared in a given assay, and therefore, such requirements could hardly be assumed to be the same for flocks reared in a different environment, with a different health status, and/or fed different feed ingredients [4].

A more suitable approach, the factorial method, accounts for inputs such as body weight (BW), body protein retention, and the efficiency of AA utilization to predict requirements irrespective of genotype, age, and environment [5]. By predicting requirements daily, based on broiler growth rates, factorial models allow poultry nutritionists to design flexible and dynamic feeding programs according to specific and multiple economic scenarios and objectives. Although more appropriate, the decision of applying a factorial approach to predict broiler AA requirements has three main concerns, which include the knowledge of daily growth rates, the amounts of post-absorbed AA partitioned to meet maintenance requirements, and the efficiency at which birds utilize AA for growth. Whereas considerable efforts have been put forwards describing broiler genotypes [6] and estimating maintenance requirements for essential AAs [7,8,9,10,11], little and controversial information regarding the efficiency of AA utilization are available in the literature. The efficiency of utilization of a given AA may be understood as the dietary fraction of such AA, which, after consumed and post-absorbed, is retained in bodily proteins. Inaccuracies when estimating the efficiency of AA utilization impact tremendously feeding programs since the bird requirements for protein gain are directly affected by such values.

Lysine and Arg are defined as basic essential AAs, whose carbon skeleton cannot be synthesized by poultry, and, which, therefore, must be provided by diets to meet bird needs for maintenance and growth. The published literature describes the efficiency of Lys utilization for broilers ranging from 0.50 to 0.81 [8,10,11]. Presumably, such discrepancies may be attributed to ongoing genetic improvements of broiler strains that may not have only increased body protein accretion rates but also improved the efficiency with which post-absorbed nutrients are utilized for protein deposition. Contrary to Lys, there is little information regarding the efficiency of Arg utilization in broilers, which consequently constrains the utilization of factorial approach to estimate requirements for such an amino acid. The focus of this research was to investigate broiler responses to SID Lys and SID Arg and, based on such responses, to estimate the efficiency with which birds utilize both AAs for growth; additionally, two factorial models to estimate broiler intakes for SID Lys and SID Arg were developed. Therefore, twelve dose–response assays were performed to estimate the efficiency of Lys and Arg utilization for male and female broiler chickens in the starter, grower, and finisher phases; based on such values, factorial models to predict broiler requirements for both AAs were developed.

## 2. Materials and Methods

All animal procedures were approved by the Animal Care and Use Committee of the Faculty of Agrarian and Veterinary Sciences of São Paulo State University, Jaboticabal, São Paulo, Brazil (protocol nº9321/18) prior to the beginning of the assays.

### 2.1. Birds Husbandry and Experimental Design

A total of 7680 Cobb 500^®^ broilers (3840 male and 3840 female) were used in six dose–response assays (three assays for SID Lys and three assays for SID Arg). In each one of the phases investigated (starter, 1–14 d; grower, 15–28 d; and finisher, 29–42 d of age), 1280 broilers (640 male and 640 female) were randomly assigned to one of sixteen experimental treatments, with four replicates of 20 birds each (64 experimental units). Experimental treatments consisted of a 2 × 8 factorial scheme (sex and 8 diets). In each assay, birds were housed in an environmentally controlled facility in 1 m × 3 m (length × width) pens covered with wood-shaving litter and equipped with nipple drinkers and hanging feeders, which provided ad libitum access to water and feed (mash form) throughout the entire experimental period. Each pen was considered as an experimental unit. The environmental temperature and the humidity were controlled according to the recommendation of the genetic strain guideline, and the photoperiod was set daily at 16 h of light and 8 h of dark. The birds used in the starter phase (1 to 14 d of age) were immediately assigned to treatments after feather-sexing. The birds used in the grower and finisher phases were reared in a different environment-controlled facility and fed diets formulated according to the genetic guideline recommendations [12]. Finally, after achieving 15 d and 29 d of age, such birds were used in the grower and partly in the finisher phase assays, respectively. Therefore, both the grower and the finisher birds did not have access to experimental feeds from other assays, and the residual effects of previous treatments were nonexistent.

### 2.2. Experimental Diets

The experimental diets were formulated using the dilution technique [13] in which a high-protein diet, limiting the studied AA (Lys or Arg), was serially diluted with a nitrogen-free diet (NFD) to obtain the desired concentrations of the referred AA (Table 1).

Except for protein and AAs, both diets were formulated to meet or exceed the nutritional recommendations described by [14]. The high-protein diet provided 1.20 times the SID Lys (Lys assays) and SID Arg (Arg assays) content recommended by [15], whereas the remaining essential AAs were provided to meet or exceed 1.40 of such recommendations. The concentration of SID Lys (Table 2) investigated ranged from 8.73 to 17.5 g Lys/kg feed in the starter phase, from 7.80 to 15.7 g Lys/kg feed in the grower phase, and 6.40 to 12.8 g Lys/kg feed in the finisher phase.

In the Arg assays, the content of SID Arg (Table 2) ranged from 9.30 to 18.7 g Arg/kg feed in the starter phase, from 8.38 a 16.8 g Arg/kg feed in the grower phase, and from 6.85 to 13.7 g Arg/kg feed in the finisher phase. An additional treatment, named “control,” was included in all the assays to confirm whether Lys and Arg were the limiting AA in the feeds used in their respective assays. The average daily gain (ADG) was the response variable evaluated for this purpose. The control diets were produced by adding crystalline Lys (L-Lys HCl, 78.5%) and Arg (L-Arg, 99%) to the diets containing the lowest concentration of both AAs to achieve the second lowest concentration of Lys and Arg under study, respectively. The data from the treatment group were only used with the purpose to investigate if the difference on the response variable was due to the studied amino acid and then removed before proceeding with further analysis.

### 2.3. Data Collection

At the beginning and the end of each assay, feed leftovers and birds were weighed to determine the average daily feed intake (ADFI) and the ADG. For those response variables, the pen average was considered for data analysis. The deposition of protein, Lys, and Arg in the feather-free body (FFB) and feathers were measured using the comparative slaughter technique, sampling birds at the beginning (six male and six female) and at the end (two birds per experimental unit) of each phase. At the beginning of starter phase, broilers were euthanized using carbon dioxide, and feathers were manually removed from the body. For the beginning of the grower and the finisher phases, birds were previously fastened for 24 h. Both tissues were freeze-dried for 72 h at −80 °C under 800 mbar of pressure (Edwards^®^ 501 Modulyo freeze dryer, Thermo Fisher, Asheville, NC, USA), grounded, and analyzed for nitrogen and for total AA content. Amino acids were analyzed (Table 3) using high-performance liquid chromatography (Brazil) as recommended by [15]. Briefly, the results of a performic acid oxidation were neutralized by sodium metabisulfite, which was then hydrolyzed using 6 N HCl for 24 h at 110 °C to release the amino acids from the protein. The ninhydrin at 570 nm was used as a standard to measure the absorption of reaction products. At the end of each phase, two birds from each experimental unit were selected according to BW, and the same procedures mentioned above were adopted. The total nitrogen content of feeds, the FFB, and the feathers were measured using the Kjeldahl method [16], and crude protein was calculated by multiplying the analyzed nitrogen by 6.25. In this case, a sample average was obtained from the mean of six birds in the reference group and two broilers per pen in the end of each assay.

### 2.4. Efficiency of Utilization and Factorial Models

The efficiency of SID Lys and SID Arg utilization (*k*) was estimated as the slope of the first-degree linear regression equation: *AAdep = a + k × AAti*, where *AAdep* (mg) is the studied AA deposition, *a* is the intercept, and *AAti* (mg) is the AA intake discounting maintenance needs reported by [8,9]. Based on the efficiency of utilization estimated, two factorial models (one for Lys and one for Arg) were adjusted to estimate broiler requirements for SID Lys and Arg. The first factorial model, named M1, was adapted from [8] and adjusted for both lysine and arginine study, as follows:

(M1) $$AAi=\left(AAT_{m}\times BW^{0.75}\right)+\left(AATD/k\right)$$

where *AAi* is the amino acid intake (mg/bird/day), *AAT_m_* is the daily intake of SID Lys for maintenance (45 mg) determined by [8] or the daily intake of SID Arg for maintenance (36 mg) determined by [9], *k* is the efficiency of Lys or Arg utilization, and *AATD* is the daily amino acid deposition in the body (mg). The daily amino acid deposition was estimated from BWG (body weight gain) according to the linear regression equation: *AATD = c + d × BWG*, where *c* is the intercept on the *Y*-axis, and *d* represents the AA deposition per unit of BWG.

A second factorial model, named M2, adapted from [4], was adjusted for both lysine and arginine study and considered the requirements for maintenance and growth of FFB and feathers separately, because the AA profile of both components are remarkably different (Table 3). The second model was set as follows:

(M2) $$\begin{array}{c} AAi=[\left(AAT_{m}\times {BP_{m}}^{0.73}\times u\right)+\left(FL\times FP\times AAT_{f}\right)]\\ +\left[\left(AAT_{b}\times BPD+AAT_{f}\times FPD\right)/k\right] \end{array}$$

where $AAT_{m}$ is the daily intake of Lys (151.2 mg) to maintain one unit of FFB protein determined by [8] or the daily intake of Arg (151.0 mg) to maintain one unit of FFB protein determined by [9] and expressed as metabolic body protein weight at maturity (${BP_{m}}^{0.73}$, kg); u is the degree of maturity (*u* = *BP*⁄$BP_{m}$, kg/kg); feather loss (*FL*) is equal to 0.01 g of feather/day [17]; *FP* is feather protein weight (g); $AAT_{f}$ (mg/g) is Lys or Arg content in feathers; $AAT_{b}$ (mg/g) is Lys or Arg content in body; and BPD and FPD (g/day) are *FFB* protein deposition (g/day) and feather protein deposition, respectively.

To obtain the inputs for each model, a Gompertz [18] function was adjusted using the estimates published by [6] (Table 4). An average broiler was generated based on the growth parameter of male and female Cobb 500^®^ chickens. The outputs obtained from the Gompertz equation such as BW, BWG, and protein deposition in the body and feathers were inputted in model M1 and model M2 to determine the Lys and Arg intake. The estimates obtained from each factorial model were compared with the requirements described by [14] and the Cobb 500^®^ guideline [12].

### 2.5. Statistical Analysis

The response variables SID AA intake and AA deposition were analyzed as two-way ANOVA using the GLM procedure of SAS 9.4 (SAS Institute Inc., Cary, NC, USA). The data were tested for ANOVA assumptions, in which the residuals were found to be normally distributed and homoscedastic between treatment groups. Variables were analyzed using the GLM procedure, accounting for two factors (dietary AA level and sex) and their interaction as the following model: Yijk = µ + αi + βj + (αβ)ij + ɛijk, where Yijk is the response variable accounting for each factor’s level (ijk), µ is the overall mean of the response variable, α is the main effect of dietary AA, β is the main effect of sex, αβ accounts for the interaction effect between factors, and ɛ is the model residue. The Dunnett’s one-tailed *t* test was used to test if the ADG of the broilers consuming the control feed was significantly greater than the broilers consuming the feed with the lowest AA level (data not presented). When the ANOVA results were significant for the interaction between factors, the effect of the SID AA level was unfolded for each sex, and the pen averages were compared using Tukey’s multiple comparison test. Additionally, polynomial contrasts were used to examine broiler response patterns to dietary levels of SID Lys and SID Arg.

To test whether the efficiency of Lys and Arg utilization differed among phases and/or between the sexes investigated, a linear regression with groups was applied to test the slope of the linear regression, the groups being phases and sexes (GenStat, VSN International, Hemel Hempstead, England). The model used for such adjustment is as follows: efficiency of utilization = constant + AA + sex + phase + AA *×* sex + AA *×* phase, where AA is the studied amino acid. Interactions were excluded from the model in case of no significance at a 0.05 probability level.

To evaluate the error of prediction from each factorial model (M1 and M2), the root mean square errors (RMSE) were calculated as follows:

(1) $$RMSE=\sqrt{\frac{\sum {\left(observed-predicted\right)}^{2}}{number\;of\;samples}}$$

## 3. Results

Regardless of the studied AA, phase and sex, broilers fed the control diet exhibited improvements in AA deposition compared with birds fed the lowest level of AA tested (AAT) (*p* < 0.05). In the starter phase, the group of broilers receiving the lowest level of SID Arg was removed from the study because they demonstrated an acute deficiency and prostration. The AA intake and the AA deposition on the body and feathers were improved with the increment of dietary SID Lys and Arg (Table 5 and Table 6). The interaction between dietary SID Lys levels and sex was observed only for the final phase, on both response variables. The interactions observed for dietary SID Arg and sex was more evident, and only the intake of SID Arg in the initial and finisher phases did not differ significantly. In the Lys assay, for male broiler chickens, Lys deposition (DLys) increased linearly until a value of 11.6, 13.1, and 8.5 g dig Lys/kg of feed (*p* < 0.01) in the starter, grower, and finisher phases, respectively. In the same phases, DLys was improved until 11.6, 11.8, and 10.7 g dig Lys/kg of feed (*p* < 0.01) for female broiler chickens. Regardless of sex, the Arg deposition (DArg) in FFB was improved at 15.6, 13.9, and 13.7 g dig Arg/kg of feed (*p* < 0.01) in the starter, grower, and finisher phases, respectively.

### 3.1. Efficiency of Utilization

Irrespective of the studied AA, no effects of sex or phase were noticed on the efficiency of utilization estimated (*p* > 0.05; Table 7). The efficiency of Lys utilization was estimated at 0.80, 0.81, and 0.81 for the starter, grower, and finisher male broilers, respectively, and at 0.78, 0.76, and 0.78 for females in the same phases. The efficiency of Arg utilization for male broiler chickens was estimated at 0.63, 0.63, and 0.62 in the starter, grower, and finisher phases, respectively, whereas females utilized Arg for growth at 0.61, 0.63, and 0.61 in the starter, grower, and finisher phase, respectively. Because interactions were non-significant, the interactions were removed from the regression model, and the estimated values for the efficiency of utilization were 0.79 for SID Lys and 0.62 for SID Arg.

### 3.2. Amino Acid Intake of Broiler Chickens

**Figure 1:**
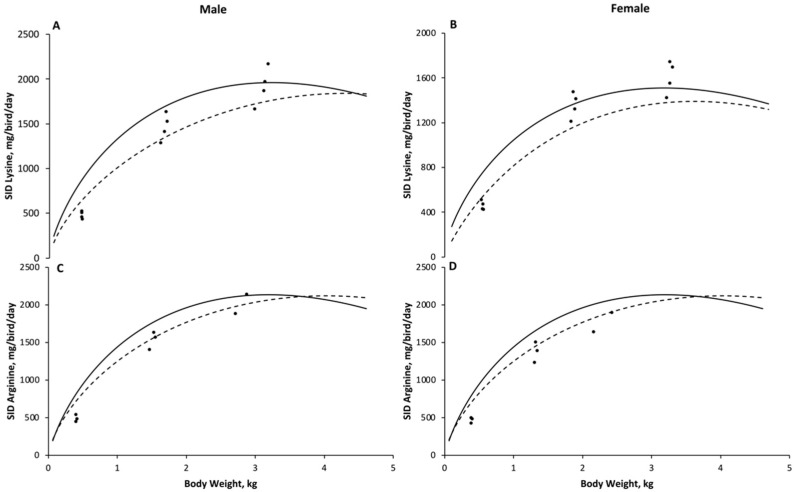
Standardized ileal digestible lysine or arginine intake estimated with model M1 (straight line) or model M2 (dashed line). The Gompertz function was used to generate the inputs for each factorial model. Observed data are represented with dots. Intake of lysine of males (**A**; number of observations = 12, root mean square error (RMSE) for M1 = 269 and M2 = 225), intake of lysine of females (**B**; number of observations = 12, root mean square error (RMSE) for M1 = 189 and M2 = 238), intake of arginine of males (**C**; number of observations = 8, root mean square error (RMSE) for M1 = 237 and M2 = 151), and intake of arginine of females (**D**; number of observations = 8, root mean square error (RMSE) for M1 = 288 and M2 = 167).

The amino acid intake predicted from the M1 model was higher than predictions obtained with model M2, and both models estimated a higher amino acid intake for males after 14-d-old when compared with the predictions for females (Figure 1).

**Figure 2:**
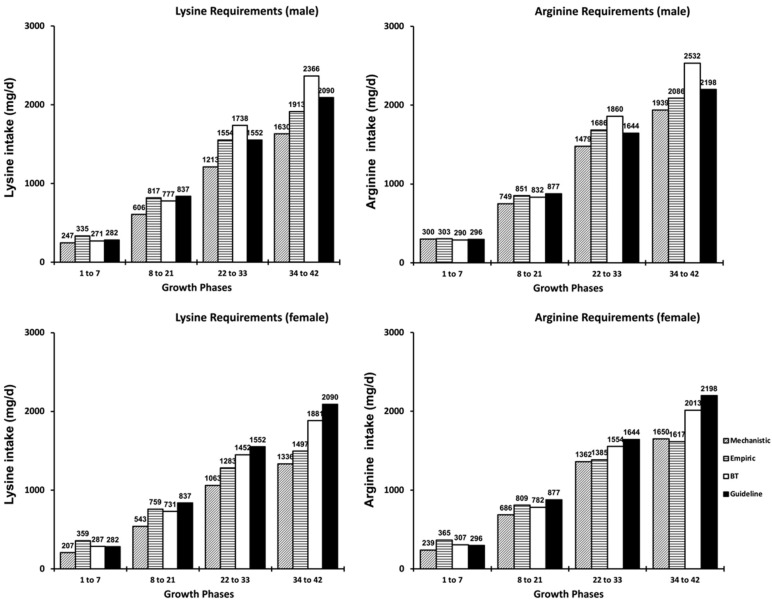
Lysine and arginine requirements of male or female broilers chickens at different growth phases established by model 1 (M1), model 2 (M2), Brazilian Tables for Poultry and Swine (BT), and Cobb 500^®^ lineage guidelines (Guideline).

For SID Lys, the M1 model predicted a superior intake than the M2 predictions, but the differences between models reduced with broilers’ age. For males, the predictions of the M1 model were 1.39 times the predictions from the M2 model in the first day, reducing to 1.15 times at day 42. For females, the predictions of the M1 model ranged from 1.91 to 1.10 times the predictions observed for the M2 model, for broilers with 1-d-old and 42-d-old, respectively. For SID Arg, the predictions from both the M1 and M2 models demonstrated a slight difference for males, ranging from 0.93 to 1.15 times the predictions from M1 related to the M2 model (Figure 1). On the other hand, a wide difference between model prediction was observed for females. The intake of Arg predicted with the M1 model was around 1.7 times higher than the predictions from M2 for a one-d-old chick, but the differences seemed to be evident until day 28, when the predictions between models became similar. The intake of SID Lys and Arg estimated by both models were compared with the recommendations described by the Cobb 500^®^ guideline [12] and Brazilian Tables for Poultry and Swine (BT) [14], as shown in Figure 2.

### 3.3. Lysine Intake of Broiler Chickens

Based on the value of efficiency of SID Lys utilization (79%) and the concentration of Lys in body protein (Table 8), broiler chickens required 94.9 mg of SID Lys per g of body protein deposited, regardless of the growth phase and sex. The M1 model estimated the male intake for SID Lys at 241, 455, 817, 1231, 1600, 1850, and 1955 mg/bird/day (RMSE = 269) and 270, 466, 759, 1064, 1313, 1465, and 1512 mg/bird/day (RMSE = 225) for females at 1, 7, 14, 21, 28, 35, and 42 days of age, respectively (Figure 1). For the same ages, according to M2, the optimum SID Lys intake was estimated at 174, 337, 606, 931, 1257, 1535, and 1730 mg/bird/day (RMSE = 189) for males and 141, 291, 543, 833, 1097, 1285, and 1377 mg/bird/day (RMSE = 238) for females.

### 3.4. Arginine Intake of Broiler Chickens

Based on the efficiency of SID Arg utilization (62%) and the concentration of Arg in body protein (Table 8), the amount of SID Arg required to deposit 1 g of body protein was 92.9 mg, irrespective of the phase and sex assessed. The SID Arg intake for male and female Cobb 500^®^ estimated by the models M1 and M2 are detailed in Table 8. The intakes for SID Arg estimated by M1 were 195, 439, 851, 1321, 1738, 2018, and 2130 mg/bird/day (RMSE = 237) for males and 267, 483, 809, 1145, 1418, 1583, and 1630 mg/bird (RMSE = 151) for females at 1, 7, 14, 21, 28, 35, and 42 days of age. The M2 model estimates of optimum SID Arg intake were 209, 413, 749, 1146, 1530, 1840, and 2038 mg/bird (RMSE = 288) for males and 157, 348, 686, 1073, 1402, 1606, and 1672 mg/bird/day (RMSE = 167) for female broiler chickens.

## 4. Discussion

The main objective of the current research was to estimate the efficiency of Lys and Arg utilization for growth in male and female broiler chickens at different growth phases and, based on such values, to model bird requirements for both AAs. Because experimental feeds were formulated using the dilution technique [13], we could assess broiler responses to a wide range of SID Lys or SID Arg doses maintaining unaltered the ratio among all the essential AAs in the feeds. Therefore, the antagonistic interactions among AAs were minimized as the concentration of AAT was gradually increased in feeds. Another advantage of this method is that the ratio between free amino acids and protein-bound amino acids is not modified regardless of the concentration of the AAT, which avoids imbalances in the pool of free amino acids in the blood and the catabolism of AAs, which, in turn, could decrease the efficiency of utilization [1,19]. In both assays, the lowest level of Lys and Arg studied corresponded to approximately 50% of the highest level, regardless of the sex and phase assessed. This wide amplitude of Lys and Arg concentration increased the magnitude of growth responses, i.e., protein and AA deposition rates, which consequently allowed estimating the efficiency of Lys and Arg utilization for growth. In the current research the more diluted the experimental diets, the poorer were the concentration of essential and non-essential AA, and consequently the lower the intake of AAs, BW, and protein deposition on FFB and feathers of broiler chickens.

Although in the current research the efficiencies of Lys utilization were numerically different, no statistical effects of age or sex were noticed on the values estimated. Consequently, the regression model without the interaction effect was used to estimate the value of 0.79 for male and female broilers throughout the entire growth period. Our outcomes support previous research findings that the efficiency of Lys utilization for growth in male broilers is unaffected by the growth phase [8]. According to such authors, the efficiency of Lys utilization for growth ranged from 0.73 to 0.79 throughout the growth period, resulting in an average value of 0.77. The values estimated in the current assay were higher than that reported by [10] who estimated the efficiency of Lys utilization for male broiler chickens from 8 to 21 days as 0.71. Sklan and Noy [11] calculated the efficiency of Lys utilization to be between 0.52 to 0.81 for males. Presumably, the discrepancies between our findings and the published literature might be attributed to methodological aspects, in this case, the feed formulation technique used to produce experimental treatments. Except [8], all the other authors mentioned above-formulated feeds based on the supplementation technique in which the studied AA was gradually added to a basal low-protein diet in its crystalline form to produce dietary treatments or to genetic improvements in broiler strains. As highlighted by [20], such a technique may increase the catabolism of the first, limiting AA and decreasing its efficiency of utilization. Another hypothesis for the greater values found in our study is the continuous genetic improvements of broiler strains. Because modern hybrids have been increasingly selected to convert feed protein into bodily protein more efficiently, a higher absorption and/or utilization of AA provided by feeds is expected for strains currently used compared with genotypes reared decades ago.

Based on Arg intake and bodily protein deposition data, we estimated the efficiency of Arg utilization for growth to be between 0.61 to 0.63 for females and 0.62 to 0.63 for males for the growth phases assessed. Similarly, to Lys assays, no statistical differences were noticed in the efficiencies of Arg utilization among the growth phases and sexes; thus, the value of 0.62 was considered for both sexes in the entire growth period. We are not aware of any published study in which the efficiency of Arg utilization for broiler chickens was measured, which makes the comparison between our outcomes and the published literature difficult. In the current research, the efficiency of Lys utilization was higher than Arg. Such outcomes were already expected and might be presumably related to the postabsorptive metabolism of each one of the studied AA. Whereas post-absorbed Lys is utilized almost in its totality for bodily protein synthesis [21], Arg is versatile and participates in several metabolic functions such as the synthesis of ornithine, polyamines, prolines, creatine, nitric oxide (NO), and citrulline, which, in turn, modulate immune and inflammatory responses and affect energy metabolism [22,23,24]. Because Arg is such a versatile AA, any physiological process, which disrupts bird homeostasis such as heat stress or immune challenges, for example, may affect its partition in post-absorbed metabolism and consequently shift its efficiency of utilization. Klasing [25] reported that AA needs can be increased seven times as the immune system is activated, due to the production of antibodies, mainly by the production of acute-phase proteins. The activation of the immune system is expected to have a significant effect on AA deposition because it is assumed that the efficiency with which protein is utilized for the purposes of the immune response is modified [26]. Under a health challenge, broilers increase the plasma NO concentrations, suggesting a shift of Arg metabolic prioritization, which may impact the prediction of efficiency utilization for protein deposition and, consequently, the requirement for growth [27,28,29]. It is essential to notice that this observation does not prove a shift in the efficiency of utilization but rather evidences that the metabolic fate of Arg is highly dependent on the health status of the broiler.

As well as for Lys, genetic selection may have also impacted the efficiency of Arg utilization. The high growth rate in modern broilers has increased the metabolic demand for oxygen. The hypoxic state, due to the limited blood supply in the breast muscle, triggers myopathies (white striping, wooden breast, and spaghetti meat) that affect the pectoralis major of fast-growing broiler chickens [30,31]. Dietary Arg can elevate plasma NO levels, a potent vasodilator molecule that can improve blood flow to the breast muscle, decreasing cellular damage [27]. There is only one physiological pathway of the production of NO, where Arg is converted into citrulline and NO stoichiometrically (1:1) by catalytic action [22,32,33]. There is evidence that NO production in healthy broiler chickens is approximately 85% lower than in infected birds (6.92 µM vs. 47.01 µM) [29]. In our study, the animals were in the ideal condition of temperature and sanitary status; thus, the efficiency of Arg utilization estimated here may not represent the Arg utilization in the worst breeding conditions, since the organism may prioritize the Arg to produce NO instead of body deposition.

Based on our findings for Lys or Arg utilization, two models were adjusted to the data: a model in which the requirements for maintenance and growth were based on BW and BWG (M1) and a second one that was more complex, which considered the differences in the AA profile of body and feathers and which expressed requirements based on body protein weight and protein deposition (M2) [4]. The main advantage of the M1 model is that the inputs necessary to predict requirements may be easily obtained from the breeder guidelines. However, it is worth recalling that AA are not required either to maintain body lipid reserves or for body lipid deposition [34]. Therefore, the prediction of AA requirements will not differ according to the amount of lipids in the body. The M2 model accounts for body and feather protein weight exclusively, and besides ignoring lipid weight when predicting requirements, it accounts for differences in the AA composition of body and feathers; it thereby partitions maintenance and growth requirements into feather-free bodies and feathers. In addition, the errors obtained with M2 were smaller than errors from M1 (Figure 1), which suggests that the principles used to develop M2, as explained above, are reasonable.

To compare the values estimated by our models with published nutritional recommendations, a four-phase feeding program was simulated (Figure 2). For the first two phases, regardless of the studied AA, the AAT intakes estimated by M1 were higher than those described by Brazilian Tables for Poultry and Swine (BT) [14] and the Cobb500^®^ guidelines [12]. From 21-days-old the intake recommendations of Lys and Arg reported in the Brazilian Tables were higher than values estimated by M1, M2, and the guidelines. A factorial approach was also provided in the BT to determine the Lys intake for broiler chickens, in which the requirement for maintenance was 70 mg/kgBW^0.75^, whereas the lysine needs for maintenance used herein was 45 mg/kgBW^0.75^ [8], which may partly explain the difference observed between both predictions. In the case of Arg, no factorial equation was provided in the BT, so the ideal ratio with lysine given was applied. The ratio between Arg:Lys intake determined by M1 and M2 were, on average, 103% and 121% for males, while, for females, this ratio was 106% and 123%, which were higher than the 107% recommended by BT and the 105% suggested by Cobb500^®^. In a recent study, it was also observed that the estimates obtained for M1 and BT were higher than those obtained from M2 [35], which, presumably, might be associated with the inputs used by models to predict requirements mainly because M2, unlike other models, does not consider the lipid fraction of BW to determine AA requirements. It is interesting to notice that no equation was reported by Cobb [12], suggesting that their recommended values are the amount necessary to achieve the performance given in the guidelines or perhaps something close to the best economic return. However, since the price of broiler production and revenues change very often, nutritionist should be able to calculate the amount of AA in the feed and adjust their profit. The models presented herein demonstrate to be a reliable method to estimate the intake for SID Lys and Arg, allowing nutritionists to rapidly change a feed formula, accommodating variations that may occur, such as an increase in ingredient prices. The M2 model estimated the SID Lys and Arg with reduced error, but the inputs were based on a feather-free body and feather protein, which are not the usual data collected in poultry farms and may prove difficult in application. Model M1 was practical because body weight and body weight gain were the inputs in the model, but nutritionists need to be aware that the errors of predictions may increase when using this model. The use of factorial models, especially the ones presented, are only possible with a correct value of efficiency of utilization, and this is, perhaps, the important contribution of this study.

## 5. Conclusions

Our findings demonstrated that the efficiency of Lys and Arg utilization was not affected by either age or sex. On average, Lys and Arg were utilized for growth at 0.79 and 0.62 times, respectively, for each unit of SID amino acid consumed. Estimating the efficiency of utilization is a first, but necessary, step towards a prediction of broiler nutritional needs, once it allows calculating the intake of SID Lys and Arg required per unit of body protein deposition. For broilers raised in a non-limiting condition, the results presented herein demonstrate that the efficiency of utilization is rather constant among age and between gender of broilers. Nonetheless, in practical situations, the AA requirement is usually expressed per unit of time, which implies that to obtain the intake of essential AA two premises need to be accounted for: the animal growth ratio and maintenance needs. Factorial models perfectly fit those premises and were used in this study to demonstrate how nutritionists can quantify the intake of SID Lys and SID Arg to achieve a specific growth. In future research, it would be valuable to validate the estimate of our models contrasting with different recommendations in a trial in which performance, carcass composition, and nitrogen retention would be measured. This research updated the efficiency of Lys utilization in modern broiler chicken genotypes, and consequently, bird requirements for such AA. The models updated in the current research allow poultry nutritionists to design dynamic feeding programs for broiler chickens with different genetic potentials and according to specific targets. Additionally, our study provides innovative knowledge regarding broilers responses to Arg intake and the efficiency with which such birds utilize Arg for growth, which, to the best of our knowledge, had not been investigated thus far.

## Figures and Tables

**Table 1:** Ingredients (g/kg) of the high-protein and nitrogen-free diets used on a dilution series to produce treatment feeds with crescent levels of lysine and arginine for broiler chickens.

Ingredients	High Protein (Arginine)	High Protein (Lysine)	Nitrogen-Free
Corn	269	279	-
Corn Starch	-	30.0	653
Soybean Meal (45%)	174	-	-
Full Fat Soybean	166	176	-
Sugar	-	-	100
Peanuts Meal	-	95.1	-
Soybean Protein Concentrate (63%)	150	246	-
Corn Gluten Meal (60%)	80.0	12.7	-
Potassium Carbonate	-	-	10.6
Soybean Oil	65.0	65.0	54.9
Dicalcium Phosphate	17.7	17.1	24.3
Limestone	8.40	9.10	8.10
Salt	4.50	4.40	3.40
Sodium Bicarbonate	-	-	2.00
DL-Methionine (99%)	6.70	7.60	-
L-Lysine HCl (78%)	7.50	3.80	-
L-Threonine (98.5%)	3.20	3.80	-
L-Tryptophan (98.5%)	0.300	0.300	-
L-Valine (96%)	2.60	3.40	-
L-Arginine (100%)	-	0.600	-
L-Isoleucine (100%)	1.20	2.00	-
L-Histidine (100%)	0.400	0.500	-
Choline Chloride (60%)	1.00	1.00	1.00
Mineral and Vitamin Premix ^1^, Additives ^2^,	3.00	3.00	3.00
Inert Filler ^3^	40.0	40.0	140
	Calculated Composition (g/kg) ^4^		
		
Crude Protein	321	311	-
AMEn ^5^ (kcal/kg)	3200	3200	3200
Digestible Lysine	20.4 (20.0)	17.5 (18.7)	-
Digestible Methionine	10.9 (10.8)	10.9 (10.7)	-
Digestible Methionine + Cystine	15.1 (15.6)	15.1 (15.1)	-
Digestible Threonine	13.4 (15.8)	13.4 (14.0)	-
Digestible Valine	15.7 (16.6)	15.7 (16.6)	-
Digestible Tryptophan	3.70	3.67	-
Digestible Arginine	18.7 (19.4)	21.8 (22.0)	-
Digestible Isoleucine	13.6 (14.7)	13.7 (14.6)	-
Digestible Leucine	27.1 (29.2)	21.8 (23.3)	-
Digestible Histidine	7.50 (8.20)	7.54 (7.70)	-
Digestible Phenylalanine + Tyrosine	25.8	23.4	-
Digestible Glycine + Serine	24.0 (27.8)	23.4 (27.6)	-
Calcium (g/kg)	9.00	9.00	9.00
Available Phosphorus (g/kg)	4.50	4.50	4.50

^1^ Inclusion of 2.00 g of premix/kg feed. Content/kg premix: beta-carotene = 6.34 mcg retinol activity equivalent; cholecalciferol = 63.9 mcg; alpha-tocopherol = 14.9 mg; menadione = 1.80 mg; thiamine = 2.00 mg; riboflavin = 4.50 mg; pyridoxine = 2.50 mg; folate = 2.00 mg; niacin = 30.0 mg; calcium pantothenate = 11.7 mg; folic acid = 0.750 mg; biotin = 0.010 mg; iron = 43.4 mg; copper = 8.56 mg; manganese = 56.0 mg; zinc = 43.4 mg; iodine = 0.560 mg; selenium = 0.340 mg. ^2^ Inclusion of 0.700 g of Salinomycin sodium (12%)/kg of feed and 0.300 g of zinc bacitracin (15%)/kg of feed. ^3^ Inclusion of 40.0 g of washed sand per kg of feed in all treatment-feeds and 100 g of rice husk per kg of nitrogen-free feed. ^4^ Values between parentheses refers to total amino acid analyzed. ^5^ Nitrogen-corrected apparent metabolizable energy.

**Table 2:** Standardized ileal digestible lysine (Lys) and digestible arginine (Arg) concentration obtained from different dilution series between high-protein (HP) and nitrogen-free (NFD) diets used in the starter, grower, and finisher phases.

Starter Phase (0 to 14 Days of Age)				Grower Phase (15 to 28 Days of Age)				Finisher Phase (29 to 42 Days of Age)			
Lys	Arg	HP	NFD	Lys	Arg	HP	NFD	Lys	Arg	HP	NFD
(g/kg)	(g/kg)	(%)	(%)	(g/kg)	(g/kg)	(%)	(%)	(g/kg)	(g/kg)	(%)	(%)
8.73	9.30	50.0	50.0	7.80	8.38	45.0	55.0	6.40	6.85	37.0	63.0
10.2	10.9	58.0	42.0	9.11	9.78	52.0	48.0	7.50	7.99	43.0	57.0
11.6	12.5	67.0	33.0	10.4	11.2	60.0	40.0	8.50	9.13	49.0	51.0
13.1	14.0	75.0	25.0	11.8	12.5	67.0	33.0	9.60	10.3	55.0	45.0
14.5	15.6	83.0	17.0	13.1	13.9	75.0	25.0	10.7	11.4	61.0	39.0
16.0	17.1	92.0	8.0	14.4	15.4	82.0	18.0	11.7	12.5	67.0	33.0
17.5	18.7	100.0	0.0	15.7	16.8	90.0	10.0	12.8	13.7	73.0	27.0
10.2 ^1^	10.9 ^1^	50.0	50.0	9.11 ^1^	9.78 ^1^	45.0	55.0	7.50^1^	7.99 ^1^	37.0	63.0

^1^ Control feeds.

**Table 3:** Protein (% of dry matter) and amino acid content (mg of amino acid/100 g of protein) of broiler chickens sampled at different ages.

Composition	Feather-Free Body				Feather			
	Sampling Age							
	1	14	28	42	1	14	28	42
Crude Protein	55.9	48.8	49.9	50.7	89.8	93.4	88.2	92.9
Amino Acid								
Lysine	5.71	6.19	7.52	7.51	1.69	1.78	1.98	1.66
Arginine	5.89	5.76	5.64	5.92	6.85	6.69	6.43	6.84
Methionine	2.00	2.01	1.98	1.99	0.368	0.414	0.424	0.241
Cystine	0.839	0.625	0.573	0.986	4.91	4.70	5.48	6.14
Threonine	4.00	3.98	3.79	4.18	4.38	4.43	4.79	5.05
Tryptophan	1.15	1.05	1.07	1.64	0.440	0.348	0.676	0.172
Valine	4.59	4.51	4.26	4.56	5.49	6.11	6.99	7.32
Isoleucine	3.69	3.79	3.64	4.08	4.07	4.08	4.65	4.94
Leucine	6.92	6.96	6.62	7.16	7.59	7.12	7.71	8.02
Glycine	6.78	7.22	6.87	7.28	7.35	6.54	6.77	7.59
Histidine	1.80	2.02	2.14	2.88	2.13	1.03	0.71	0.65
Phenylalanine	3.88	3.79	3.67	3.75	5.77	4.78	4.88	4.67
Serine	4.33	3.72	3.53	3.95	11.6	10.4	11.4	13.1
Tyrosine	3.05	2.91	2.72	2.64	4.67	3.05	2.93	2.29
Glutamic acid	12.5	13.0	12.7	12.1	9.90	9.86	11.0	7.70
Alanine	5.50	5.86	5.67	6.33	3.21	3.47	3.83	4.58
Aspartic acid	7.76	7.76	7.55	8.74	6.82	6.00	6.28	7.00
Proline	-	-	-	5.11	-	-	-	10.9

**Table 4:** Gompertz ^1^ function parameters of body and protein growth of broiler chickens.

Male/Female			
Parameters ^2^	Body Weight	Body Protein	Feather Protein
Wm (kg)	8.416/6.558	1.557/1.024	0.266/0.205
B (g/day)	0.038/0.037	0.031/0.035	0.040/0.053
t* (kg)	42.7/40.6	52.5/45.7	40.1/34.0

^1^ Gompertz equation: Wm*exp(−exp(−B*(X−t*))) where X is age. ^2^ Wm, mature weight; B, deposition rate; t*, time (days) where the growth is maximal. Data from Vargas (2020).

**Table 5:** Effects ^1^ of standardized ileal digestible levels (SID Lys) in intake (iLys) and deposition on body and feathers (DLys) on male and female broiler chickens in the starter, grower, and finisher phases.

SID Lysine ^2^		Starter Phase (1 to 14 Days of Age)				Grower Phase (15 to 28 Days of Age)				Finisher (29 to 42 Days of Age)			
		ILys (mg/Day)		DLys (mg/Day)		ILys (mg/Day)		DLys (mg/Day)		ILys (mg/Dday)		DLys (mg/Day)	
		Male	Female	Male	Female	Male	Female	Male	Female	Male	Female	Male	Female
8.73/7.8/6.4		283	274	228	239	830	698	647	466	636 ^i^	614 ^i^	42.5 ^e^	151 ^e^
10.2/9.11/7.5		348	358	285	299	1036	889	811	634	1020 ^h^	884 ^h^	405 ^cd^	228 ^d^
11.6/10.4/8.5		411	383	335	344	1213	1049	977	737	1510 ^f^	1283 ^g^	925 ^ab^	576 ^c^
13.1/11.8/9.6		438	426	359	365	1285	1211	1030	851	1663 ^de^	1424 ^fg^	1079 ^ab^	564 ^c^
14.5/13.1/10.7		460	433	359	327	1412	1321	1176	910	1868 ^bc^	1554 ^eg^	1126 ^a^	835 ^b^
16.0/14.4/11.7		506	474	377	353	1527	1412	1126	918	1973 ^b^	1697 ^de^	1159 ^a^	871 ^b^
17.5/15.7/12.8		523	514	384	340	1637	1476	1069	811	2169 ^a^	1747 ^cd^	1051 ^ab^	846 ^b^
SEM ^3^		13.96		17.8		21.8		45.8		42.7		102	
Main Factors													
SID Lys	8.73/7.8/6.4	279 ^e^		233 ^c^		763 ^g^		559 ^e^		625		96.5	
	10.2/9.11/7.5	353 ^d^		292 ^b^		962 ^f^		723 ^d^		952		316	
	11.6/10.4/8.5	397 ^c^		340 ^a^		1131 ^e^		857 ^c^		1396		751	
	13.1/11.8/9.6	432 ^bc^		362 ^a^		1248 ^d^		940 ^bc^		1544		821	
	14.5/13.1/10.7	446 ^b^		343 ^a^		1366 ^c^		1043 ^a^		1711		981	
	16.0/14.4/11.7	490 ^a^		368 ^a^		1470 ^b^		1022 ^ab^		1835		1015	
	17.5/15.7/12.8	519 ^a^		359 ^a^		1556 ^a^		983 ^ab^		1961		948	
Sex	Male	424		332		1277		976		1548		807	
	Female	409		324		1151		773		948		551	
ANOVA *p*-value ^4^	SID Lys	<0.01		<0.01		<0.01		<0.01		<0.01		<0.01	
	Sex	0.0478		0.217		<0.01		<0.01		<0.01		<0.01	
	Interaction	0.75		0.131		0.304		0.693		<0.01		<0.01	
Polynomial Contrasts *p*-value	Linear	<0.001		<0.001		<0.001		<0.001		<0.001	<0.001	<0.001	<0.001
	Quadratic	0.025		0.086		0.001		0.086		0.025	0.001	0.025	<0.001

^1^ Means in the same column with different letters differed by Tukey’s test. ^2^ Values separated by a slash represent the standardized ileal digestible lysine (SID Lys) supplementation for each growing phase. ^3^ Pooled standard error of the mean. ^4^ For the F test, the degrees of freedom were 11 for the numerator and 36 for the denominator, given a critical F value of 2.07 for a 0.05 probability test.

**Table 6:** Effects ^1^ of standardized ileal digestible levels Arg (SID Arg) in intake (iArg) and deposition on body and feathers (DArg) on male and female broiler chickens in the starter, grower, and finisher phases.

SID Arginine ^2^		Starter Phase (1 to 14 Days of Age)				Grower Phase (15 to 28 Days of Age)				Finisher (29 to 42 Days of Age)			
		IArg (mg/Day)		DArg (mg/Day)		IArg (mg/Day)		DArg (mg/Day)		IArg (mg/Day)		DArg (mg/Day)	
		Male	Female	Male	Female	Male	Female	Male	Female	Male	Female	Male	Female
-/8.38/6.85		-	-	-	-	571 ^i^	530 ^i^	249 ^hi^	224 ^i^	786	653	305 ^de^	247 ^e^
10.9/9.78/7.99		234	207	224 ^de^	95.0 ^f^	748 ^gh^	692 ^h^	292 ^hi^	291 ^hi^	900	747	331 ^de^	268 ^e^
12.5/11.2/9.13		309	273	291 ^cd^	142 ^ef^	873 ^f^	830 ^fg^	338 ^ghi^	398 ^fgh^	1066	922	341 ^de^	301 ^de^
14/12.5/10.3		400	387	398 ^c^	202 ^def^	1077 ^e^	1006 ^e^	488 ^efg^	510 ^def^	1267	1024	469 ^cde^	337 ^de^
15.6/13.9/11.4		453	428	510 ^b^	261 ^d^	1407 ^c^	1237 ^d^	757 ^abc^	655 ^cde^	1511	1297	614 ^bcd^	532 ^bcde^
17.1/15.4/12.5		488	484	655 ^a^	257 ^d^	1537 ^ab^	1394 ^c^	834 ^ab^	679 ^bcd^	1887	1641	860 ^b^	569 ^bcde^
18.7/16.8/13.7		502	546	679 ^a^	253 ^de^	1635 ^a^	1508 ^bc^	853 ^a^	685 ^abc^	2143	1900	1237 ^a^	769 ^bc^
SEM ^3^		16.8		32.4		23.5		47.5		32.6		68.1	
Main Factors													
SID Arg	-/8.38/6.85	-		-		558		253		720 ^g^		276	
	10.9/9.78/7.99	221 ^e^		159		720		291		823 ^f^		300	
	12.5/11.2/9.13	291 ^d^		216		851		368		994 ^e^		321	
	14/12.5/10.3	393 ^c^		300		1041		499		1145 ^d^		1028	
	15.6/13.9/11.4	440 ^bc^		386		4322		706		1404 ^c^		573	
	17.1/15.4/12.5	486 ^ab^		456		1483		756		1764 ^b^		714	
	18.7/16.8/13.7	524 ^a^		466		1571		769		2021 ^a^		1003	
Sex	Male	405		459		1126		544		1366		594	
	Female	380		202		1028		492		1169		432	
ANOVA *p*-value ^4^	SID Arg	<0.01		<0.01		<0.01		<0.01		<0.01		<0.01	
	Sex	0.014		<0.01		<0.01		<0.01		<0.01		<0.01	
	Interaction	0.858		<0.01		<0.01		<0.01		0.309		0.024	
Polynomial Contrasts *p*-value	Linear	<0.001		<0.001	<0.001	<0.001	<0.001	< 0.001	<0.001	<0.001		<0.001	<0.001
	Quadratic	0.024		0.81	0.001	0.572	0.873	0.401	0.041	<0.001	<0.001	0.001	0.020

^1^ Means in the same column with different letters differed by Tukey’s test. ^2^ Values separated by a slash represent the standardized ileal digestible arginine (SID ARG) supplementation for each growing phase. ^3^ Pooled standard error of mean. ^4^ For the F test, the degrees of freedom were 11 for the numerator and 36 for the denominator, given a critical F value of 2.07 for a 0.05 probability test.

**Table 7:** Efficiency of lysine and arginine utilization for male and female broiler chickens, and the equation of amino acid deposition (AAd) in the function of intake (AAi) adjusted to estimate the efficiency of utilization.

Age	Efficiency of Lysine Utilization		Efficiency of Arginine Utilization	
	Female	Male	Female	Male
1 to 14	0.78	0.8	0.61	0.63
15 to 28	0.76	0.81	0.63	0.63
29 to 42	0.78	0.81	0.61	0.62
Average	0.79		0.62	
*p*-value phase	0.954		0.963	
*p*-value sex	0.512		0.244	
Linear equation	AAd = 23.8(± 94.2) + 0.789(± 0.256) × AAi		AAd = −30.9(± 13.6) + 0.623(± 0.024) × AAi	

**Table 8:** Digestible lysine and arginine requirements for Cobb 500^®^ broilers estimated by factorial models.

AGE (day)	Estimated Growth ^1^						Lysine Intake (mg/day)		Arginine Intake (mg/day)	
	BW (kg)	BWG (kg/day)	BPW (kg)	BPD (g/day)	FPW (kg)	FPD (g/day)	Model 1 ^2^	Model 2 ^3^	Model 1 ^4^	Model 2 ^5^
					Male					
									
1	0.060	0.010	0.010	1.71	0.002	0.430	241	174	195	209
7	0.170	0.030	0.020	3.28	0.006	0.940	455	337	439	413
14	0.420	0.050	0.060	5.88	0.016	1.77	817	606	851	749
21	0.850	0.080	0.110	9.01	0.031	2.66	1231	931	1321	1146
28	1.46	0.100	0.180	12.2	0.053	3.39	1600	1257	1738	1530
35	2.20	0.110	0.280	14.9	0.078	3.81	1850	1535	2018	1840
42	3.01	0.120	0.390	16.7	0.105	3.88	1955	1730	2130	2038
					Female					
									
1	0.080	0.010	0.010	1.41	0.001	0.210	270	141	267	157
7	0.200	0.030	0.020	2.85	0.003	0.710	466	291	483	348
14	0.440	0.050	0.050	5.21	0.012	1.76	759	543	809	686
21	0.820	0.060	0.090	7.91	0.028	2.94	1064	833	1145	1073
28	1.33	0.080	0.160	10.4	0.052	3.75	1313	1097	1418	1402
35	1.91	0.090	0.240	12.2	0.079	3.96	1465	1285	1583	1606
42	2.53	0.090	0.330	13.1	0.106	3.67	1512	1377	1630	1672

^1^ BW, body weight; BWG, body weight gain; BPW, body protein weight; BPD, body protein deposition; FPW, feather protein weight; FPD, feather protein deposition. ^2^ Lys = (45 × BW^0.75^) + ((44.8 + 12,025 × BWG)/0.79). ^3^ Lys = ((151.2 × BPW^0.73^ × (BPW/BPm)) + (0.01 × FPW × 18)) + ((75 × BPD + 18 × FPD)/0.79). ^4^ Arg = (36 × BW^0.75^) + ((−8.86 + 10,842 × BWG)/0.62). ^5^ Arg = ((151 × BPW^0.73^ × (BPW/BPm)) + (0.01 × FPW × 67)) + ((58 × BPD + 67 × FPD)/0.62).

## Data Availability

The data presented in this study are available on request from the corresponding author. The data are not publicly available due to privacy.
